# Paclitaxel-Induced Epidermal Alterations: An In Vitro Preclinical Assessment in Primary Keratinocytes and in a 3D Epidermis Model

**DOI:** 10.3390/ijms23031142

**Published:** 2022-01-20

**Authors:** Paula Montero, Javier Milara, Martín Pérez-Leal, Cristina Estornut, Inés Roger, Alejandro Pérez-Fidalgo, Celia Sanz, Julio Cortijo

**Affiliations:** 1 Department of Pharmacology, Faculty of Medicine, University of Valencia, 46010 Valencia, Spain; cristina.estna@gmail.com (C.E.); irola3@gmail.com (I.R.); Celia.Sanz@uv.es (C.S.); julio.cortijo@uv.es (J.C.); 2 Biomedical Research Networking Centre on Respiratory Diseases (CIBERES), Health Institute Carlos III, 28029 Madrid, Spain; 3 Pharmacy Unit, University General Hospital Consortium, 46014 Valencia, Spain; 4 Faculty of Health Sciences, Universidad Europea de Valencia, 46010 Valencia, Spain; Martin.perez@universidadeuropea.es; 5 Department of Medical Oncology, University Clinic Hospital of Valencia, 46010 Valencia, Spain; japfidalgo@msn.com; 6 Biomedical Research Networking Centre on Cancer (CIBERONC), Health Institute Carlos III, 28029 Madrid, Spain; 7 INCLIVA Biomedical Research Institute, 46010 Valencia, Spain; 8 Health Sciences, Pre-Departmental Section of Medicine, Jaume I University of Castellon, 12071 Castellon, Spain; 9 Research and Teaching Unit, University General Hospital Consortium, 46014 Valencia, Spain

**Keywords:** paclitaxel, epidermis, NHEK, 3D epidermis model

## Abstract

Paclitaxel is a microtubule-stabilizing chemotherapeutic agent approved for the treatment of ovarian, non-small cell lung, head, neck, and breast cancers. Despite its beneficial effects on cancer and widespread use, paclitaxel also damages healthy tissues, including the skin. However, the mechanisms that drive these skin adverse events are not clearly understood. In the present study, we demonstrated, by using both primary epidermal keratinocytes (NHEK) and a 3D epidermis model, that paclitaxel impairs different cellular processes: paclitaxel increased the release of IL-1α, IL-6, and IL-8 inflammatory cytokines, produced reactive oxygen species (ROS) release and apoptosis, and reduced the endothelial tube formation in the dermal microvascular endothelial cells (HDMEC). Some of the mechanisms driving these adverse skin events in vitro are mediated by the activation of toll-like receptor 4 (TLR-4), which phosphorylate transcription of nuclear factor kappa B (NF-κb). This is the first study analyzing paclitaxel effects on healthy human epidermal cells with an epidermis 3D model, and will help in understanding paclitaxel’s effects on the skin.

## 1. Introduction

Paclitaxel is an anticancer drug, extracted from the bark of the Pacific yew tree Taxus brevifolia [1]. It was first approved in 1992 by the US Food and Drug Administration (FDA) for the treatment of advanced ovarian cancer and since then, it has been used in several cancers such as breast cancer, endometrial cancer, non-small-cell lung cancer, bladder cancer, cervical carcinoma, and AIDS-related Kaposi sarcoma [2]. Paclitaxel exerts its anticancer activity through its microtubule stabilization properties, which impair the dissociation of microtubules during mitosis. Therefore, paclitaxel disrupts mitosis and blocks cell cycle progression, leading to cell death [1,2,3]. The mechanisms by which paclitaxel leads to apoptosis are not clearly understood. However, several signaling pathways have been described. Depending on paclitaxel concentration, it can lead to apoptosis through the induction of Raf-1 activation, which is responsible for apoptotic control, or under the influence of p53 and p21, which also regulate proliferation and apoptosis [4]. Paclitaxel has been shown to exert its mechanism of action activating other signal-transduction pathways associated with proapoptotic signaling. Paclitaxel is a ligand to toll-like receptor 4 (TLR4); after binding to TLR4, it can trigger the MyD88, mitogen-activated protein kinase (MAPK) and transcription of nuclear factor kappa B (NF-κB) pathways, which result in the release of cytokines, such as interleukin-1 (IL-1), interleukin-6 (IL-6), and interleukin-8 (IL-8) [5]. Amongst the transcriptional factors activated, NF-κB plays an important role in coordinately controlling apoptosis [6]. Paclitaxel, related to MAPK, modulates the regulatory proteins of the BCL2 family, and is involved in programmed cell death [7,8,9]; treatment with paclitaxel activates the c-Jun N-terminal kinase (JNK), which is also associated with apoptosis [10,11].

Dermatological adverse events are frequent in patients under paclitaxel treatment. However, these events are often neglected in clinical practice and the true incidence is not known. The evidence of the skin adverse effects caused by paclitaxel are described in case reports. Amongst the adverse events described, erythematous rashes, inflamed skin, macules, papules, pustules and scaling, swelling of hands and feet, oedema and dorsal hand-foot syndrome can be found. The paclitaxel-induced rash is often found on warm sites prone to trauma, such as the folds, contact areas, or under dressing. Pigmentary changes in sun-exposed areas, alopecia, and nail changes have also been described [12,13,14,15,16,17,18,19]. These effects are generally mild to moderate, classified by Common Terminology Criteria for Adverse Events (CTCAE) as grade 1 or 2 in severity. When the severity is grade 3 or higher, it is often caused by a toxic and non-immunoallergic mechanism. Therefore, they are usually dose-dependent, and sometimes necessitate transient dose interruptions, reductions, or termination of the treatment. Most evidence of these adverse events comes from case reports and oncology studies, but the mechanisms driving paclitaxel skin adverse events have not been well described [18]. Although the mechanisms of paclitaxel-induced skin alterations have not been described, a few reports examine its effects on keratinocytes: in proliferating human hair follicle matrix keratinocytes, paclitaxel induces extensive mitotic defects and apoptosis [20,21] and also induces an apoptotic response in transformed HaCat keratinocytes paclitaxel [22]. Further, cutaneous biopsies from skin lesions of patients under paclitaxel treatment show atypical keratinocytes and abundant apoptotic bodies throughout the epidermis layers [23,24]. Other studies in zebrafish larvae showed that paclitaxel promotes epithelial damage and induces keratinocyte-specific gene upregulation [25]. These findings suggest that paclitaxel skin adverse effects might be mediated by its effects on epithelial keratinocytes. Of note, studies on keratinocyte monolayer cultures can lack the physiological functions of the stratified epithelium and could misinterpret the results obtained in preclinical studies. Thereby, various three-dimensional (3D) skin equivalents reproducing in vivo conditions have been developed for pharmacologic and toxicologic in vitro testing as an alternative to animal models [26,27]. One of these models is characterized by the growth of keratinocytes on a feeder layer of lethally irradiated 3T3 fibroblasts. The feeder layer supports and maintains keratinocyte colony growth and stratification [28,29], producing a 3D model that is compatible with autologous and allogenic transplantation [30,31].

Paclitaxel maximum plasma concentrations achieved are dose-related, for instance, single-dose intravenous administration at 135–350 mg/m^2^ produces a mean plasma concentration of 0.23 to 10 μM. Of note, exposure to paclitaxel is higher in tumor tissue compared with other tissues [32], but despite its beneficial effects on cancer, paclitaxel also damages healthy tissues, including the skin. To our knowledge, the direct effects of paclitaxel on primary human keratinocytes remain elusive, and the mechanisms that promote keratinocyte alterations are yet to be elucidated. The previously described observations of paclitaxel in hair follicle keratinocytes and skin biopsies, along with the lack of investigation on the effects of paclitaxel (0.3, 3 and 30 μM) in keratinocytes, lead us to investigate its molecular effects on primary human keratinocytes and in a 3D epidermis model.

## 2. Results

### 2.1. Non-Cytotoxic Doses of Paclitaxel Induce Inflammation in a 3D Epidermis Model

**Figure 1:**
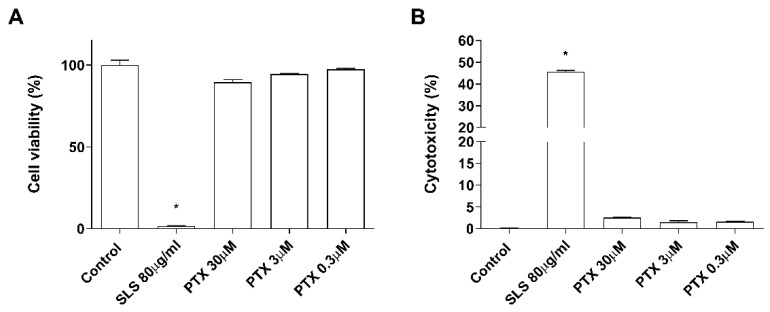
Paclitaxel does not modify cell viability and cytotoxicity. NHEK cells were incubated for 24 h with increasing paclitaxel concentrations. (**A**) Paclitaxel, at the concentrations assayed did not show alterations on cell viability measured by the MTT assay (**B**) nor in the cytotoxicity measured by LDH assay. Results are expressed as mean ± standard deviation of three independent experiments (*n* = 3). Multiple comparisons analysis of variance (ANOVA) was followed by the post hoc Bonferroni test. * *p* < 0.05 vs. control. PTX: paclitaxel. SLS: sodium lauryl sulfate.

**Figure 2:**
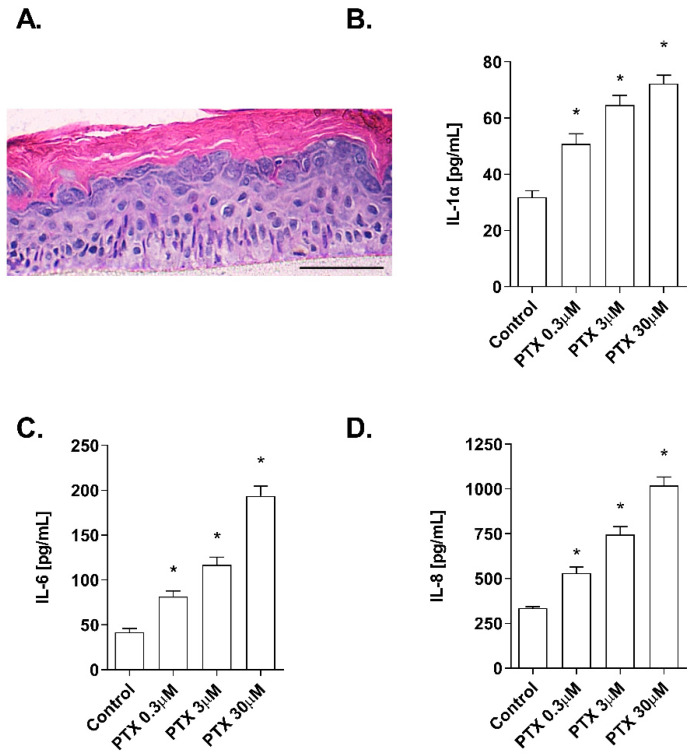
Paclitaxel induces a dose-dependent inflammatory cytokine release in a 3D epidermal model. (**A**) Paraffin section from the 3D epidermis model stained with hematoxylin and eosin. Scale bar 100 µM. The 3D epidermal model was incubated for 24 h with increasing paclitaxel concentrations. (**B**) IL-1α, (**C**) IL-6, and (**D**) IL-8 levels were measured by ELISA. Results are expressed as mean ± standard deviation of three independent experiments (*n* = 3). Multiple comparisons analysis of variance (ANOVA) was followed by the post hoc Bonferroni test. * *p* < 0.05 vs. control. PTX: paclitaxel.

The effects of paclitaxel on cell viability were examined in NHEK cells. Incubation with the positive control SLS demonstrated that both assays were sensitive to changes in viability and cytotoxicity. Treatment with increasing doses of paclitaxel (0.3–30 µM) for 24 h was safe for NHEK keratinocytes as it did not induce significant viability reduction and LDH release. The mean viability percentages were 86.53 ± 3.5%, 94.46 ± 0.8%, and 97.35 ± 1.085% at concentrations 30 µM, 3 µM, and 0.3 µM, respectively (Figure 1A). The LDH release was lower than 2.5% in all doses examined and was not significant compared to the control (Figure 1B). The stratification of the 3D epidermis cell model was confirmed by the hematoxylin-eosin staining as shown in Figure 2A. Keratinocytes were distributed into the principal epidermis layers. The basal, spinous, and granular layers are present in the reconstructed model and its terminal differentiation resulted in the presence of the stratum corneum, analogously to the epidermal in vivo structure of healthy skin. Since IL-1α, IL-6, and IL-8 are known as skin inflammation molecular markers [33,34,35], and these markers have been found to be upregulated by paclitaxel in some cancer cell lines [7,11,36], we analyzed whether paclitaxel could mediate an inflammatory response in the 3D epidermis model through the induction of such cytokines. Incubation of the 3D model with paclitaxel induced a significant dose-dependent release of IL-1α, IL-6, and IL-8 (Figure 2B–D).

### 2.2. Paclitaxel-Induced Oxidative Stress Response

**Figure 3:**
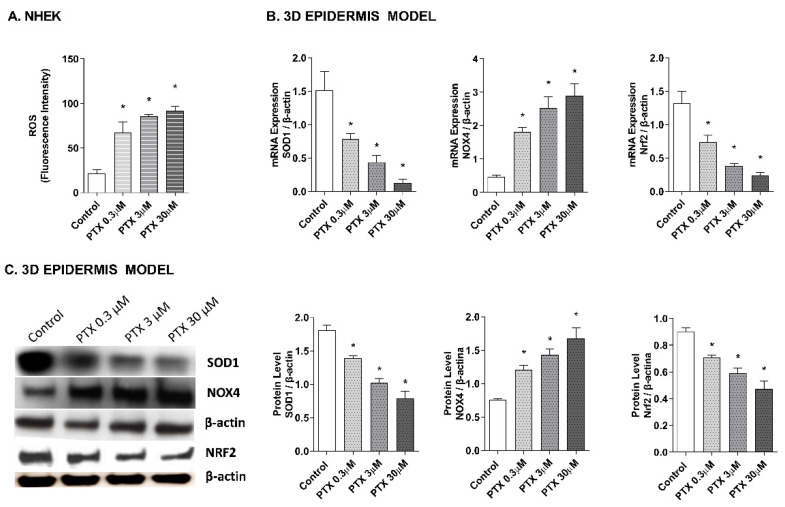
Paclitaxel induces a dose-dependent oxidative stress response in normal human epidermal keratinocytes (NHEK) cells and in a 3D epidermal model. (**A**) NHEK cells were incubated for 4 h with increasing paclitaxel concentrations. Quantification of reactive oxygen species (ROS) levels measured by the H_2_DCF-DA assay. Data are expressed as reactive oxygen species (ROS) DCF relative fluorescence units. (**B**) 3D epidermal model tissues were incubated for 24 h with increasing paclitaxel concentrations. SOD1, Nrf2 and NOX4 mRNA levels were measured by real-time PCR. Data are expressed as 2^−ΔCt^. (**C**) 3D epidermal model tissues were incubated for 24 h with increasing paclitaxel concentrations. SOD1, Nrf2 and NOX4 protein levels were analyzed by western blotting. Quantification was performed by densitometry and normalized to β-actin. Results are expressed as mean ± standard deviation of three independent experiments (*n* = 3). Multiple comparisons analysis of variance (ANOVA) was followed by the post hoc Bonferroni test. * *p* < 0.05 vs. control. PTX: paclitaxel.

As it has been proposed that the apoptotic effects of paclitaxel may be mediated by its capacity to induce the release of reactive oxygen species (ROS) [25,37,38], the effect of paclitaxel on intracellular ROS levels was analyzed in NHEK cells and in a 3D epidermis model. As shown in Figure 3A, exposure to paclitaxel doses of 0.3, 3, and 30 µM for 4 h, caused a significant increase in ROS production. To assess which molecules might be taking part in the oxidant induction of paclitaxel, gene, and protein expression of nuclear factor erythroid-2-related factor 2 (Nrf2), superoxide dismutase (SOD1), and NADPH oxidase 4 (NOX4) were analyzed in the 3D epidermal model. Nrf2 is a transcription factor that regulates the endogenous antioxidant defense, SOD1 is a ROS scavenging gene and NOX4 is one of the primary enzymatic sources of ROS. Treatment of this model with paclitaxel for 24 h induced a decrease in the mRNA expression of SOD1 and Nrf2 in a dose-dependent manner (Figure 3B). The same incubation time induced the upregulation of NOX4 in all paclitaxel doses (Figure 3B). Figure 3C shows that incubating the 3D epidermis model for 24 h with paclitaxel produced the same response in the protein expression as in the mRNA expression: A concentration-dependent decrease in both SOD1 and Nrf2 protein expression and a concentration-dependent increase in NOX4.

### 2.3. Paclitaxel-Induced Apoptosis

**Figure 4:**
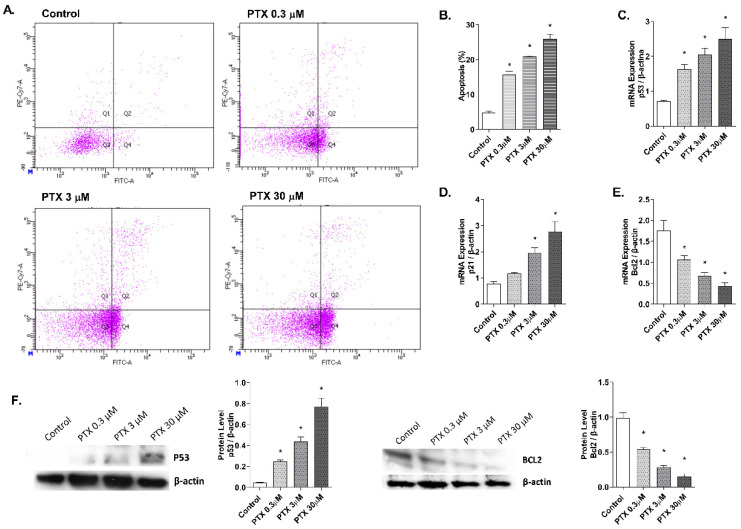
Paclitaxel induces apoptosis in normal human epidermal keratinocytes (NHEK) cells. (**A**) NHEK cells were incubated for 24 h with increasing paclitaxel concentrations. Apoptosis was measured by flow cytometric analysis. (**A**) Representative plots for each paclitaxel concentration are displayed. (**B**) Apoptosis plots were analyzed by FlowJo software (TreeStar Inc., Ashland, OR, USA). Results are expressed as the mean apoptosis percentage of annexin-positive and propidium iodide-negative cells, which represent early apoptotic cells. (**C**–**E**) 3D epidermal model tissues were incubated for 24 h with increasing paclitaxel concentrations. P53, p21, and BCL2 mRNA levels were measured by real-time PCR. Data are expressed as 2^−ΔCt^. (**F**) 3D epidermal model tissues were incubated for 24 h with increasing paclitaxel concentrations. P53 and BCL2 protein levels were analyzed by Western blotting. Quantification was performed by densitometry and normalized to β-actin. Results are expressed as mean ± standard deviation of three independent experiments (*n* = 3). Multiple comparisons analysis of variance (ANOVA) was followed by the post hoc Bonferroni test. * *p* < 0.05 vs. control. PTX: paclitaxel.

Detection of annexin V-FITC by flow cytometry was used to analyze the apoptosis percentage induced by paclitaxel in NHEK cells (Figure 4A,B). Keratinocytes incubation with paclitaxel for 24 h induced an increase in cellular apoptosis. Representative propidium iodide versus annexin V-FITC plots are shown in Figure 4A for each condition. Higher doses of paclitaxel induced significantly higher apoptosis rates, reaching up to 25.8 ± 2.9% apoptosis at the highest dose 30 µM (Figure 4B). To analyze the apoptosis molecular markers in the 3D epidermis model, gene and protein expression of p53, p21, and BCL2 were measured (Figure 4C–E). 24 h of paclitaxel incubation reduced BCL2 mRNA expression while p53 and p21 were upregulated. All markers showed a dose-dependent modulation and statistically significant variations. Protein levels of BCL2 and p53 were also analyzed by Western blot and showed a dose-dependent increase in the case of p53, while protein levels of BCL2 decreased significantly (Figure 4F).

### 2.4. Paclitaxel-Targeted Angiogenesis

**Figure 5:**
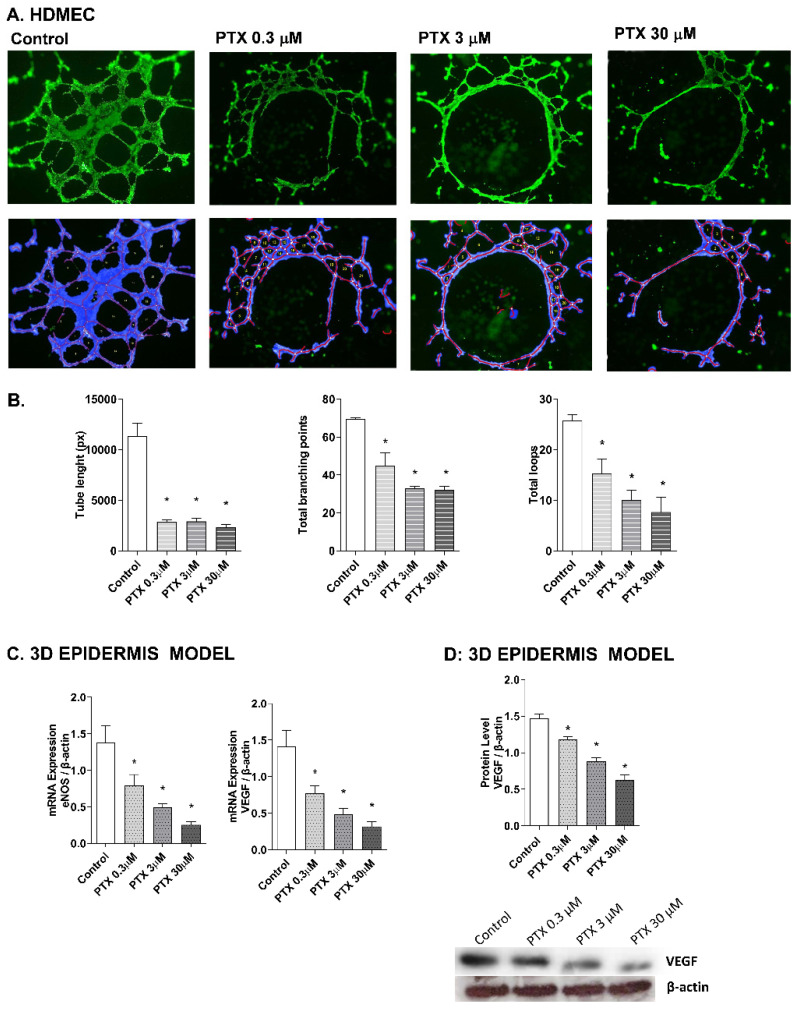
Paclitaxel inhibits endothelial tube formation in human dermal microvascular endothelial cells (HDMEC) and reduces eNOS and VEGF expression in the 3D epidermis. (**A**) HDMEC were incubated with increasing paclitaxel concentrations for 16 h, and angiogenesis was analyzed by the endothelial tube formation assay. Representative images of the tubular structures formed are displayed. Top images show the green, fluorescent calcein staining. Bottom images show the overlay generated by WimTube^TM^ software (Onimagin Technologies SCA, Córdoba, Spain), in which each color represents a structure: blue represents the covered area, red the tubes, white the branching points and yellow the number of loops. (**B**) Quantitative evaluation of morphological features of the capillary-like network structure. Tube length, total branching points and total loops after treating HDMEC with increasing paclitaxel concentrations. The analysis was performed using WimTube^TM^ software (Onimagin Technologies SCA, Córdoba, Spain). (**C**) 3D epidermal model tissues were incubated for 24 h with increasing paclitaxel concentrations. eNOS and VEGF mRNA levels were measured by real-time PCR. Data are expressed as 2^−ΔCt^. (**D**) In vitro 3D epidermal model was incubated for 24 h with increasing paclitaxel concentrations. VEGF protein levels were analyzed by Western blotting. Quantification was performed by densitometry and normalized to β-actin. Results are expressed as mean ± standard deviation of three independent experiments (*n* = 3). Multiple comparisons analysis of variance (ANOVA) was followed by the post hoc Bonferroni test. * *p* < 0.05 vs. control. PTX: paclitaxel.

Paclitaxel has a strong anti-angiogenic activity in cancer cells through the suppression of the vascular endothelial growth factor (VEGF) expression, which plays a main role in the growth of new blood vessels, by activating the endothelial nitric oxide synthase (eNOS) [39,40,41]. To evaluate these events in the skin, the effect of paclitaxel on endothelial tube formation was examined in human dermal microvascular cells. Representative images for each condition are shown in Figure 5A. HDMECs in the control condition formed capillary-like structures. However, incubation with paclitaxel for 16 h showed an impairment of angiogenesis in all doses. The analysis was performed by measuring the significant decrease of the tube length, total branches and total loops created by HDMECs in the gel matrix after paclitaxel incubation (Figure 5B). The molecular markers of angiogenesis, VEGF and eNOS, were also evaluated in the 3D epidermis model. After paclitaxel treatment, VEGF and eNOS mRNA expression were significantly reduced in a dose-dependent manner (Figure 5C). The same decrease was induced by paclitaxel in VEGF protein levels (Figure 5D).

### 2.5. NF-κB Transcription Factor Activation by Paclitaxel

**Figure 6:**
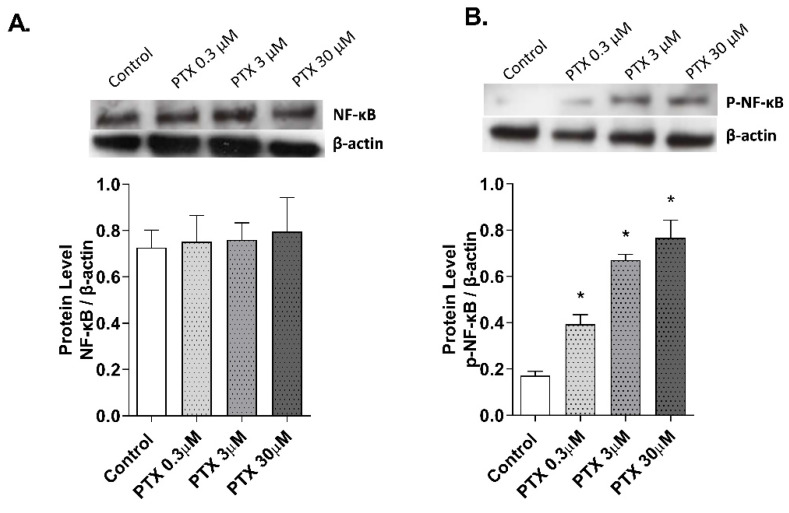
Paclitaxel activates the transcription factor NF-κB in a 3D epidermal model. The 3D epidermis was incubated for 1 h with increasing paclitaxel concentrations. (**A**) NF-κB and (**B**) p-NF-κB protein levels were analyzed by Western blotting. Quantification was performed by densitometry and normalized to β-actin. Results are expressed as mean ± standard deviation of three independent experiments (*n* = 3). Multiple comparisons analysis of variance (ANOVA) was followed by the post hoc Bonferroni test. * *p* < 0.05 vs. control. PTX: paclitaxel.

The effect of paclitaxel on NF-κB activation was evaluated in the 3D epidermis model. The 3D model was incubated for 1 h with paclitaxel and both NF-κB unphosphorylated (Figure 6A) and phosphorylated (Figure 6B) forms were analyzed by Western blot. While the unphosphorylated form of the protein remained stable after incubation with all paclitaxel concentrations, NF-κB phosphorylation increased at all doses.

### 2.6. TLR4 Mediates Paclitaxel Effects on Human Keratinocytes

**Figure 7:**
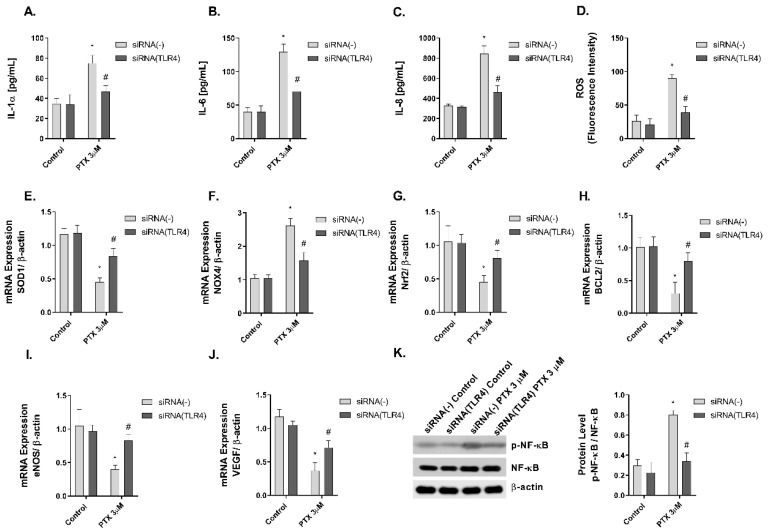
The effect of paclitaxel modulating inflammation, oxidative stress, apoptosis angiogenesis, and p-NF-κB is reduced in siRNA-TLR4 transiently transfected keratinocytes. Normal human epidermal keratinocytes (NHEK) were transiently transfected with control siRNA (-) or siRNA-TLR-4 and incubated for 24 h with paclitaxel 3 µM. (**A**–**C**) IL-1α, IL-6 and IL-8 supernatant levels were measured by ELISA. (**D**) Reactive oxygen species (ROS) were measured using H_2_DCF-DA assay in NHEK stimulated with paclitaxel for 4 h. (**E**–**J**) The expression of SOD1, NOX4, Nrf2, BCL2, eNOS, and VEGF was measured by real-time PCR. Data are expressed as 2^−ΔCt^. (**K**) NHEK cells were incubated for 1 h with paclitaxel concentrations. NF-κB and p-NF-κB protein levels were analyzed by Western blotting. Quantification was performed by densitometry and normalized to NF-κB/β-actin. Results are expressed as mean ± standard deviation of three independent experiments (*n* = 3). Multiple comparisons analysis of variance (ANOVA) was followed by the post hoc Bonferroni test. * *p* < 0.05 vs. siRNA (-) Control. # *p* < 0.05 vs. siRNA (-) PTX 3 µM. PTX: paclitaxel.

Previous evidence indicates that paclitaxel is a ligand to TLR4, which is expressed on innate immune cells, including macrophages [5,42]. However, there is no evidence in human skin. In this work, NHEK cells were transiently transfected with siRNA (-) control and siRNA-TLR4 to reduce TLR4 expression. The stimulation of NHEK cells with paclitaxel 3 µM on pro-inflammatory IL-1α, IL-6, and IL-8 cytokine release, and ROS production including SOD1, NOX4, and Nrf2 was significantly inhibited in cells transfected with siRNA-TLR4 (Figure 7A–G). The effects of paclitaxel reducing the anti-apoptotic protein BCL2 were reduced in siRNA-TLR4 treated cells (Figure 7H). The siRNA-TLR4 transfection also abrogated the effects of paclitaxel on eNOS and VEGF expression (Figure 7I,J) and reduced the phosphorylation of NF-κB in NHEK cells (Figure 7K).

## 3. Discussion

Paclitaxel’s skin adverse effects have been described from a clinical point of view and the mechanisms underlying these events have not been described thoroughly. The effects of paclitaxel on the cellular mechanisms triggering its toxicity have been studied predominantly on malignant cells [43]. To date, few studies have examined these effects in normal cells. Therefore, we wanted to investigate in this study paclitaxel in primary keratinocytes and in a 3D epidermal model, to further comprehend the mechanisms triggering its skin alterations. This is the first study analyzing paclitaxel effects on both models, showing that paclitaxel induced inflammation, oxidative stress generation, apoptosis, and angiogenesis inhibition through the activation of TLR4 and NF-κB pathways. There is a limitation of the study to be mentioned. The feeder layer that supports the epidermal growth and differentiation of the keratinocytes in the 3D model is made by BALB/3T3 fibroblasts. There is a minor possibility that the presence of fibroblasts could affect the measurements performed in this study. However, due to the substantial difference in cell number between keratinocytes and fibroblasts, and the fact that the feeder layer is irradiated and therefore, growth-arrested, we considered that the contribution of fibroblasts to the results are minor and will not significantly bias our data.

We first examined the cytotoxic effects of paclitaxel within the clinically achievable plasma concentrations, ranging from 0.3 to 30 µM, on NHEK cells. None of the concentrations reduced cell viability significantly, nor induced LDH release. These concentrations were innocuous to keratinocytes and therefore were selected for further experiments. To validate the reconstruction of the 3D epidermis model, the hematoxylin–eosin staining demonstrated the development of a fully differentiated epidermis. The 3D epidermis model was used as a mimicker of a healthy epidermis to evaluate the molecular modulation induced after treatment with the clinically achievable plasma concentrations of paclitaxel.

As paclitaxel-induced inflammation may be a trigger in the development of skin adverse effects, we analyzed the expression of cytokines IL-1α, IL-6, and IL-8 in the 3D epidermis model. IL-1 is constitutively produced by keratinocytes in the stratum corneum [33] and released as a primary response to various stimuli. It also induces the release of secondary mediators, such as IL-6 and IL-8 [44]. IL-6 stimulates keratinocyte proliferation and is studied in diseases associated with epidermal hyperplasia and in wound healing. IL-8 promotes dendritic cell migration and the recruitment of monocytes and neutrophils after external stimuli, as key steps in the initiation phase of skin inflammation. Additionally, keratinocyte production of IL-8 has been observed in autoimmune-mediated diseases [35,45]. The levels of IL-1α, IL-6, and IL-8 released by the 3D epidermis model were increased after paclitaxel treatment. While there is a lack of information that relates IL-1α to paclitaxel, previous works have described that paclitaxel induces the upregulation of IL-6 in ovarian cancer cells through the TLR4–NF-κB cascade [7]. Further, plasma levels of IL-6 and IL-8 were increased in patients with breast cancer after paclitaxel administration [46]. Paclitaxel has been shown to activate IL-8 transcriptionally in ovarian carcinoma cells [47] and increases IL-8 synthesis in a subset of human lung carcinoma cell lines through an NF-κB-dependent mechanism [36]. Other authors have related paclitaxel to the upregulation of IL-8 through the kinase JKN in a human ovarian cancer cell line [11]. These findings show that paclitaxel can upregulate different cytokines in patients and cancer cell lines, in agreement with the results obtained in this report. However, this is the first study reporting paclitaxel-induced increase of IL-1α, IL-6, and IL-8 in a 3D epidermis model.

Regarding apoptosis, paclitaxel induces the activation of the kinase Raf1, which is responsible for the apoptotic control through the suppression of BCL2, an antiapoptotic protein [41,48]. In the absence of Raf1 activity, it can induce apoptosis under the influence of the proteins, p53 and p21 [4]. The tumor suppressor protein p53 regulates proliferation and apoptosis. In normal cells, DNA damage increases levels of p53, which then triggers a cell cycle arrest mediated by the p21 protein, to promote DNA damage repair mechanisms or apoptosis [4,49]. As expected, we observed an increase in apoptosis in NHEK keratinocytes after treatment with paclitaxel. In agreement with these results, paclitaxel has been shown to cause apoptosis in hair matrix keratinocytes [20,21]. Additionally, paclitaxel modulates the expression of BCL2 and increases p53 expression levels [50,51]. Here, we showed that treatment with paclitaxel in the 3D epidermis model reduced the BCL2 gene and protein levels and increased gene and protein expression of p21 and p53. These results show that the effects derived from the antineoplastic action of paclitaxel also occur in epidermal cells and its apoptotic effects in the skin might be driven by different signaling pathways, as shown by the modulation of BCL2, p53 and p21.

It has been proposed that the apoptotic effects of paclitaxel may be mediated by its capacity to induce the release of ROS [25,37,38]. However, the relationship of oxidative stress to the overall mechanism of paclitaxel is not well established. Therefore, we analyzed the oxidative stress response induced by paclitaxel in keratinocytes and the 3D epidermis model. Paclitaxel has been shown to induce ROS in multiple cell lines, such as the human breast cancer line [37,52,53], A549 cells [53], stromal fibroblasts [54], and endothelial cells [38]. Similarly, our results showed that exposing NHEK cells to increasing doses of paclitaxel increased ROS production. We then examined, in the 3D epidermis model, the gene and protein expression of SOD1, Nrf2, and NOX4, proteins involved in the regulation of ROS, and used as markers in the study of cellular oxidation. The SOD1 enzyme acts by dispersing oxygen superoxide and hydrogen peroxide in the cell and the transcription factor Nrf2 plays an important role in regulating the transcription of antioxidant proteins. On the other hand, NOX4 is a key factor in the intracellular homeostasis of oxidation reactions and is recognized as one of the main ROS producers [50,55,56,57]. Treatment with paclitaxel in the 3D epidermis model led to a reduction in SOD1 and Nrf2 gene and protein expression, which implies that paclitaxel impairs its antioxidant response, as well as in cancer-associated fibroblasts [58]. Contrarily, NOX4 was upregulated by paclitaxel in the 3D epidermal model, consistently with the increase in ROS production seen on NHEK cells. Of note, NOX4 upregulation associated with an ROS increase has also been described by other authors in human breast cancer lines [37,52,53].

Paclitaxel accumulates in endothelial cells [59] and exhibits a strong anti-angiogenic activity in cancer cells through the suppression of VEGF expression [39,40]. VEGF takes part in the angiogenesis signaling pathway by activating the endothelial nitric oxide synthase eNOS [60], which has reduced expression after paclitaxel treatment in endothelial cells [41,51]. Physiological levels of nitric oxide (NO) are required to maintain the normal functioning of cells, including keratinocytes. NO is vital as a signaling molecule regulating multiple epidermal functions, including keratinocyte proliferation and differentiation, apoptosis, migration, and oxidative stress, as well as cytokine production [61]. NO is produced by eNOS, that is expressed in human keratinocytes in a similar way that VEGF [61,62]. In this work, we examined if the anti-angiogenic activity of paclitaxel could happen as well in the skin, by analyzing endothelial tube formation in primary human dermal microvascular endothelial cells (HDMEC). Treatment with paclitaxel in HDMEC produced a decrease in the generation of the endothelial tube. It affected the number of branches and loops formed, and the total length of the tube network. In the 3D epidermis model, VEGF and eNOS expression was reduced as well as the protein expression of VEGF. From these results, we speculated that reduced levels of eNOS imply low levels of physiological NO. This could affect the proper epidermis permeability and wound healing, and induce an imbalance of pro-inflammatory cytokines [61], as we have seen in our results. As some authors propose that paclitaxel anti-angiogenic properties may be regulated by ROS production in endothelial cells [38], we can infer that elevated levels of oxidative stress in skin keratinocytes might also drive the anti-angiogenic properties in HDMEC. Then, our results obtained in skin cells regarding the modulation of angiogenesis by paclitaxel, correlate with those seen on cancer cell lines and show that, since angiogenesis is a process closely related to skin alterations [63], it may be taking part in paclitaxel’s skin adverse effects.

After paclitaxel treatment, activation of NF-κB transcription factor has been shown to play an important role in the regulation of inflammation, apoptosis, and cell cycle progression [64]. This transcription factor has also been associated with paclitaxel-induced ROS production and angiogenesis [38]. Given that, we decided to examine the activation of the transcription factor in a 3D epidermal model. Treatment of this model with paclitaxel induced the phosphorylation of the factor NF-κB, which indicated its activation, and its possible implication in driving the cellular processes investigated in this report. Between the different activators of NF-κB, TLR4 plays an important role in the innate immune response. The activation of TLR4 triggers different molecular pathways including JNK, P38 and NF-κB [65]. Previous reports have shown that paclitaxel can activate TLR4 in macrophages and dendritic cells, mimicking the effects of lipopolysaccharide secreting inflammatory cytokines [5,66]. TLR4 is expressed in human keratinocytes and its activation has been related to inflammatory, oxidative, and anti-proliferative effects [67], showing antineoplastic effects in cutaneous squamous cell carcinoma [68]. In this work, we showed novel evidence on the effects of paclitaxel activating TLR4 and promoting NF-κB phosphorylation to induce keratinocyte inflammation, oxidative stress, apoptosis, and dermal antiangiogenic activity. The reduction of TLR4 expression by siRNA-TLR4 partially abrogated the cellular effects induced by paclitaxel in keratinocytes. Currently, the dermatological adverse effects of paclitaxel have been described from a clinical perspective, but the knowledge about their cellular and molecular mechanisms is lagging. There is limited literature in which the effects of paclitaxel in healthy keratinocytes are explained. However, our results present novel evidence of the effects of paclitaxel on skin. This report shows that paclitaxel activates TLR-4 and promotes NF-κB phosphorylation, which results in the increase of oxidative stress, inflammation, and apoptosis, and the reduction of angiogenesis. These events could explain the direct skin side effects of paclitaxel in healthy skin, although the interplay between the different cellular processes and the associated signaling pathways are yet to be discovered.

## 4. Materials and Methods

### 4.1. Cell Culture and 3D Epidermis Model Reconstruction

Cellular experiments were performed in normal human epidermal keratinocytes (NHEK) (PromoCell, Heidelberg, Germany) and primary human dermal microvascular endothelial cells (HDMEC) (PromoCell, Heidelberg, Germany). NHEK were cultured in keratinocyte growth medium-2 (KGM-2), supplemented with SupplementMix and CaCl_2_ (60 µM) (Promocell, Heidelberg, Germany). HDMEC were cultured in endothelial cell growth basal medium-2 (Lonza, Basel, Switzerland). All cell lines were maintained in a humidified 5% CO_2_ atmosphere at 37 °C.

3D epidermis cell models were reconstructed using the BALB/3T3 feeder-layer technique adapted from Mak et al. [69] and Arnette et al. [28]. In brief, 10^6^ BALB/3T3 fibroblasts (Lonza, Basel, Switzerland) were seeded on collagen-coated Millicell inserts (Millicell-CM 12 mm, transparent Biophore Membrane; Millipore CORP., Bedford, UK) and placed into 6-well plates (Corning Incorporated; Corning, NY, USA). Fibroblasts were cultured for 2 days in 1 mL Dulbecco’s Modified Eagle Medium (DMEM, high glucose) (Gibco®, Life Technologies Corporation, Madrid, Spain) supplemented with 10% fetal calf serum (FCS) (Gibco®, Life Technologies Corporation, Madrid, Spain) and added to the apical and dorsal side of the insert. When fibroblasts reached 60–70% confluence, the monolayer was irradiated with UV light at 0.048 mW for 1 h with UVACUBE 400 (Honle UV Technology, Gilching, Germany) to establish the feeder layer. Then, primary adult epidermal keratinocytes (192627, Lonza, Basel, Switzerland) were seeded at a density of 0.5 × 10^6^ cells/cm^2^. Cultures were grown at 37 °C and 95% air/5% CO_2_ until approximately 60% confluency and then were switched to KGM-2, supplemented with SupplementMix and CaCl_2_ 60 µM (Promocell, Heidelberg, Germany) until confluent. Confluent cultures were raised to the air–liquid interface and cultured for 21 days until epidermal stratification was achieved. To validate the stratification, histological analysis was performed after 21 days. The reconstructed epidermis tissues were fixed with 10% formalin solution, dehydrated, and embedded in paraffin. Six-micrometer-thick sections were cut and stained with hematoxylin–eosin. Random photographs were taken of each sample with a Leica DM6000B microscope (Leica Biosystems; Wetzlar, Germany).

### 4.2. Cell Viability and Cell Death Assay

NHEK cells were cultured in a 96-well plate (Corning Incorporated, New York, NY, USA) until reaching 60% confluence. Then, cells were incubated for 24 h with different concentrations of paclitaxel (30, 3, 0.3, and 0.03 μM). Paclitaxel was dissolved in dimethyl sulfoxide (DMSO) and then, dilutions were performed in cell culture medium. The final concentration of DMSO in the culture did not exceed 0.001%. Cell viability 24 h after removal of the treatment was determined using the MTT assay. 1 mg/mL MTT solution was added to the treated cells and incubated for 3 h at 37 °C. After incubation, cells were washed with phosphate-buffered saline (PBS), and DMSO was added for 10 min to dissolve the formazan precipitate. Absorbance was measured at 572 nm using the plate reader Infinite M200 (Tecan Group Ltd., Männedorf, Switzerland). Data were normalized to control values. 24 h after treatment, the cell death assay was performed by measuring lactate dehydrogenase (LDH) release in the medium using the commercially available LDH cytotoxicity assay kit (Thermo Fisher Scientific, Waltham, MA, USA), following the manufacturer’s instructions. Absorbance was measured at 490 nm using the plate reader Infinite M200 (Tecan Group Ltd., Männedorf, Switzerland). LDH contents were normalized to the maximum LDH release. Sodium lauryl sulfate (SLS) was used in both experiments as a positive control at 80 μg/mL.

### 4.3. Cytokine Determination by ELISA

3D epidermal model tissues were incubated for 24 h with different paclitaxel concentrations (0.3, 3, and 30 μM). After incubation, the culture medium was collected for each condition. IL-8, IL-6, and IL-1α cytokine levels were analyzed using commercially available Quantikine^®^ ELISA kits (R&D Systems, Madrid, Spain) according to the manufacturer’s protocol.

### 4.4. DCF Fluorescence Measurement of Reactive Oxygen Species

2′, 7′-dichlorodihydrofluorescein diacetate (H_2_DCF-DA) (Invitrogen, Thermo Fisher Scientific, Waltham, MA, USA) is a cell-permeable compound. When intracellular ester hydrolysis is oxidized to fluorescent 2′, 7′-dichlorofluorescein (DCF) by O2 and H_2_O_2_, it can therefore be used to monitor intracellular generation of ROS. To quantify ROS levels, NHEK were cultured in 96 black cell culture plate with transparent bottom, washed twice with PBS and incubated for 30 min with 50 µM H_2_DCF-DA diluted in Opti-MEM. Then, cells were stimulated with different paclitaxel concentrations (0.3, 3 and 30 μM) for 4 h. Fluorescent intensity was measured using a microplate spectrophotometer (Victor 1420 Multilabel Counter, PerkinElmer, Madrid, Spain) at excitation and emission wavelengths of 485 and 528 nm. Results were expressed as ROS fluorescence intensity, which indicate DCF fluorescence in relative fluorescence units.

### 4.5. Real-Time RT-PCR and siRNA Experiments

3D epidermal model tissues were incubated for 24 h with different paclitaxel concentrations (0.3, 3, and 30 μM). After incubation, total RNA was extracted using TRIzol^®^ Reagent (Invitrogen, Thermo Fisher Scientific, Waltham, MA, USA) following the manufacturer’s instructions. Reverse transcription was performed in 500 ng of total RNA with a TaqMan reverse transcription reagents kit (Applied Biosystems, Thermo Fisher Scientific, Waltham, MA, USA). cDNA was amplified with specific primers and probes predesigned by Applied Biosystems for SOD1 (Hs00533490_m1), Nrf2 (Hs00975961_g1), NOX4 (Hs01379108_m1), p21 (Hs01040810_m1), p53 (Hs01034249_m1), VEGF (Hs00900055_m1), BCL2 (Hs04986394_s1) and eNOS (Hs01574665_m1) in a QuantStudio™ 5 Real-Time PCR System, using a universal master mix (Applied Biosystems, Thermo Fisher Scientific, Waltham, MA, USA). β-actin (Hs01060665_g1) was used as an endogenous control. The mean value of the replicates for each sample was calculated and expressed as the cycle threshold (Ct). The level of gene expression was then calculated as the difference (ΔCt) between the Ct value of the target gene and the Ct value of β-actin. The fold changes in the target gene mRNA levels were designated 2^−ΔCt^.

Small interfering RNA (siRNA) experiments were carried out in NHEK. The scrambled siRNA control (siRNA (-)) was purchased from Ambion (Huntingdon, Cambridge, UK; catalogue No. 4390843). TLR4 gene-targeted siRNA (siRNA-TLR4) (identification No. s14195) was designed by Ambion. Cells were transfected with siRNA (50 nM) in serum and antibiotic-free medium. After 6 h, the medium was aspirated and replaced with medium containing serum for a further 42 h before cell stimulation. The transfection reagent used was lipofectamine-2000 (Invitrogen, Paisley, UK; catalogue No. 11668-027) at a final concentration of 2 µg/mL. Transiently silenced NHEK were incubated with paclitaxel at 3 μM for 24 h.

### 4.6. Western Blotting Analysis

3D epidermal model tissues were incubated for 24 h with different paclitaxel concentrations (0.3, 3 and 30 μM) and transiently silenced NHEK were incubated with paclitaxel at 3 μM for 24 h. After incubation, protein extraction was performed incubating samples with lysis buffer (1 M HEPES, 4 M NaCl, 0.5 M EDTA, and 0.1 M EGTA) supplemented with the protease inhibitory cocktail complete™ and phenyl–methyl–sulfonyl fluoride (PMSF) (Roche Diagnostics; Indianapolis, IN, USA). Total protein concentration was quantified using the BCA protein assay kit (Thermo Fisher Scientific, Waltham, MA, USA). Protein electrophoresis was performed to separate proteins according to their molecular weight. 12 μg of denatured proteins along with Rainbow™ molecular weight marker (Sigma–Aldrich, St. Louis, MO, USA) were loaded into Mini-PROTEAN^®^ polyacrylamide gels TGX™ (Bio-Rad, Herts, UK), by application of 100 V during 1 h. Proteins were transferred from the gel to a nitrocellulose membrane Trans-Blot^®^ Turbo™ Transfer Pack, using the Trans-Blot^®^ Turbo ™ Transfer System (Bio-Rad Laboratories; Herts, UK). Then, membranes were incubated with 5% bovine serum albumin (BSA) for 2 h and labelled overnight at 4 °C, with various primary antibodies. The secondary antibody was incubated for 1 h at room temperature. The primary antibodies and concentrations used were the following: SOD1 1:2000 (ab16831, Abcam, Cambridge, UK), Nrf2 1:1000 (ab89443, Abcam, Cambridge, UK), NOX4 1:1000 (NB100-58849, Novus Biologicals, Cambridge, UK), p53 1:1000 (ab131442, Abcam, Cambridge, UK), BCL2 1:500 (NB100-92142, Novus Biologicals, Cambridge, UK), VEGF 1:2000 (ab46154, Abcam, Cambridge, UK), NF-κB 1:2000 (ab16502, Abcam, Cambridge, UK) and p-NF-κB 1:1000 (ab86299, Abcam, Cambridge, UK). To normalize results, β-actin antibody 1:7000 (A1978, Sigma–Aldrich, St. Louis, MO, USA) was used as housekeeping control. Signal visualization of proteins was carried out by incubating the membranes with chemiluminescence reagents (ECL Plus; Amersham GE Healthcare, Buckinghamshire, UK). Densitometry of films was performed using the Image J 1.42q software (USA). Results of target protein expression are expressed as the percentage of the densitometry of the endogenous controls β-actin.

### 4.7. Apoptosis

NHEK cells were seeded on 96-well plates and incubated for 24 h with different paclitaxel concentrations (0.3, 3 and 30 μM). Apoptosis was measured using a commercially available Annexin V-FITC apoptosis detection kit (ab14085, Abcam, Cambridge, UK). Cells were detached and collected along with the supernatant and incubated with annexin V-FITC in a final concentration of 3 μg/mL for 15 min. Then, annexin V binding buffer was added and prior to flow cytometric analysis, propidium iodide was added at 5 μg/mL. Flow cytometric analysis was performed by a BD LSRFortessa™ X-20 flow cytometer (BD Biosciences; San Jose, CA, USA). A minimum of 10,000 cells per sample were analyzed with Flow-Jo standard software (TreeStar Inc., Ashland, OR, USA).

### 4.8. Angiogenesis

Determination of changes in the angiogenesis process was analyzed using the commercial endothelial tube formation assay kit (Cell Biolabs, San Diego, CA, USA). HDMEC cells were seeded on the plates coated with the matrigel provided by the kit. Incubation with paclitaxel at different concentrations (0.3, 3, and 30 μM) was performed for 16 h, following the manufacturer’s instructions, to allow the endothelial tube formation. After incubation, cells were stained with a calcein-acetoxymethyl-based staining solution (calcein-AM) for 30 min. Images were captured by fluorescence microscopy (Spectral Leica TCS SP2 microscope, Leica Biosystems; Wetzlar, Germany). The morphological features were quantitatively measured to characterize the capillary-like tube structure using the software WimTube™(Onimagin Technologies SCA, Córdoba, Spain). The software analysis provides the tube length, branching points, loops, and cell-covered area. Besides that, detailed overlay images are provided in which all branching points, tubes, and cells are noticeable.

### 4.9. Statistical Analyses

Results from cellular in vitro experiments were expressed as mean ± standard error (SE) of n experiments. Normal distribution for each data set was confirmed by the Kolmogorov—Smirnov test. Statistical analysis was carried out by multiple comparisons analysis of variance (ANOVA) followed by Bonferroni post hoc test. *p* < 0.05 was considered statistically significant.
